# A Critical Assessment of Diagnostic Criteria for the Tall Cell Subtype of Papillary Thyroid Carcinoma—How Much? How Tall? And When Is It Relevant?

**DOI:** 10.1007/s12022-023-09788-8

**Published:** 2023-10-21

**Authors:** John Turchini, Talia L. Fuchs, Angela Chou, Loretta Sioson, Adele Clarkson, Amy Sheen, Leigh Delbridge, Anthony Glover, Mark Sywak, Stan Sidhu, Anthony J. Gill

**Affiliations:** 1 Anatomical Pathology, Douglass Hanly Moir Pathology, Macquarie Park, NSW 2113 Australia; 2 Discipline of Pathology, Macquarie Medical School, Macquarie University, NSW 2109 Australia; 3 Sydney Medical School, The University of Sydney, Sydney, 2006 Australia; 4 Cancer Diagnosis and Pathology Group, Kolling Institute of Medical Research, St Leonards, NSW 2065 Australia; 5 NSW Health Pathology, Department of Anatomical Pathology, Royal North Shore Hospital, NSW Health Pathology, St Leonards, NSW 2065 Australia; 6 Endocrine Surgical Unit, Royal North Shore Hospital, St Leonards, NSW 2065 Australia

**Keywords:** Papillary thyroid carcinoma, Tall cell subtype, Thyroid carcinoma

## Abstract

Tall cell papillary thyroid carcinoma (TC-PTC) is considered adverse histology. However, previous studies are confounded by inconsistent criteria and strong associations with other adverse features. It is therefore still unclear if TC-PTC represents an independent prognostic factor in multivariate analysis and, if it does, what criteria should be employed for the diagnosis. We retrospectively reviewed 487 PTCs from our institution (where we have historically avoided the prospective diagnosis of TC-PTC) for both the height of tall cells (that is if the cells were two, or three, times as tall as wide) and the percentage of tall cells. On univariate analysis, there was significantly better disease free survival (DFS) in PTCs with no significant tall cell component (< 30%) compared to PTCs with cells two times tall as wide (*p* = 0.005). The proportion of tall cells (30–50% and > 50%) was significantly associated with DFS (*p* = 0.012). In a multivariate model including age, size, vascular space invasion, and lymph node metastasis, the current WHO tall cell criteria, met by 7.8% of PTCs, lacked statistical significance for DFS (*p* = 0.519). However, in the subset of tumours otherwise similar to the American Thyroid Association (ATA) guidelines low-risk category, WHO TC-PTC demonstrated a highly significant reduction in DFS (*p* = 0.004). In contrast, in intermediate to high-risk tumours, TC-PTC by WHO criteria lacked statistical significance (*p* = 0.384). We conclude that it may be simplistic to think of tall cell features as being present or absent, as both the height of the cells (two times versus three times) and the percentage of cells that are tall have different clinical significances in different contexts. Most importantly, the primary clinical significance of TC-PTC is restricted to PTCs that are otherwise low risk by ATA guidelines.

## Introduction

The tall cell subtype of papillary thyroid carcinoma (TC-PTC) has long been considered a poor prognostic category of PTC [1–13]. In general, the diagnosis of TC-PTC requires that a significant proportion of the neoplastic cells be taller than they are wide. However, the precise criteria applied have varied over time and in different institutions. In their entity defining description, Hawk and Hazard originally described TC-PTC as a PTC in which at least 30% of the neoplastic cells are at least twice as tall as wide [14]. During the following two decades, most publications kept the twice as tall as wide criteria [15–18]. However by 1996, some publications began to use height criteria of three times as tall as wide [19]. In the WHO 2004 system, the 3:1 ratio was endorsed [20]. Subsequent publications used either the 3:1 or 2:1 cut-off [5, 21–24] and in the WHO 2017 system the tall cell component was required, somewhat ambiguously, to be *‘two to three times as tall as they are wide’* [25]. Similarly, the proportion of cells which must be ‘tall’ has varied over time with cut-offs of 10%, 30%, 50%, and 70% being proposed and used by different authors [1–32].

The current World Health Organization (WHO) 2022 criteria unambiguously defines TC-PTC as a tumour with more than 30% of cells being 3 times tall as wide [1, 12]. However, given the very different criteria used by different groups, it is not surprising that the historical reported incidence of TC-PTC in different series has varied widely—from 1.3% to 12% of PTCs in different institutions [1–32]; and there is strong evidence that the pathological diagnosis of TC-PTC is subject to poor interobserver concordance [32]. A confounding feature in assessing the clinical significance of tallness is that, because it is so well established as an adverse prognostic factor; once the diagnosis of TC-PTC is made, patients may be offered more aggressive therapy and this may make it hard to retrospectively assess the independent clinical significance of different criteria for TC-PTC.

Our experience confirms that different institutions certainly have different tendencies to invoke the diagnosis of TC-PTC. Historically (that is prior to 2012), at our two tertiary referral centres in Sydney, Australia, we had only rarely used the diagnosis of TC-PTC. Whilst this was out of step with many centres, it reflected our experience that TC-PTCs commonly already show a variety of other features associated with aggressive behaviour. Therefore, we, in agreement with at least some others, felt that the presence of tall cells commonly does not alter the treatment options for our patients because most TC-PTCs are also associated with other pathological or clinical features suggesting a high risk of aggressive behaviour [32, 33].

As a result of our institutions’ historical bias against making a pathological diagnosis of TC-PTC, when we review our database of PTCs treated in our institution from 1985 to 2012, only 14 of 2053 (0.68%) of resected PTCs were recorded as TC-PTC—much lower than in any other published series. Of course, this can only be because during that historical period we had not labelled as tall cell subtype many tumours that would be considered TC-PTC by many, if not most, pathologists by current criteria; and if they were re-reviewed by different criteria many would be considered TC-PTC.

Regardless of the relative merits of applying a low or high threshold to the diagnosis of TC-PTC, this large cohort of patients with PTCs that may show tall cell features (but were not treated differently to other tumours as they were not prospectively identified as TC-PTC) offers the unique opportunity to retrospectively assess the validity of different criteria for the diagnosis of TC-PTC and investigate questions such as what cut-offs for both height and proportion should be employed to make the diagnosis of TC-PTC and whether the presence of TC-PTC is so strongly associated with other adverse features that it may not have independent clinical significance in all circumstances.

We therefore sought to use this unique cohort to critically assess criteria in current use for the diagnosis of TC-PTC.

## Methods

We searched the computerized database of the department of Anatomical Pathology, Royal North Shore Hospital, for all PTCs from 1985 to 2012 for which we had both archived blocks/slides and at least 10 years of confirmed clinical follow available [34, 35]. Non-invasive follicular thyroid neoplasm with papillary like nuclear features (NIFT-P), and poorly differentiated/insular/anaplastic carcinoma arising from PTC were excluded.

**Fig. 1:**
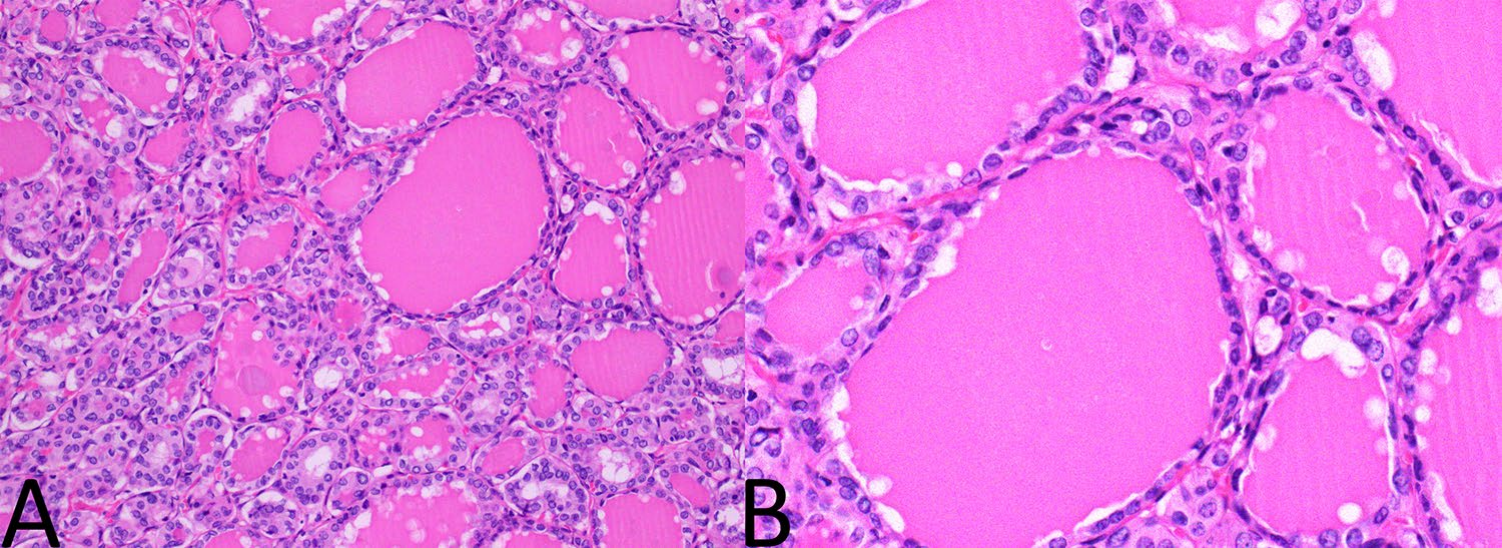
Papillary carcinoma without tall cell features. **A** Follicular architecture (H&E, 100x). **B** Cells demonstrating nuclear features of papillary carcinoma without tall cell features or increased cell height (H&E, 400x)

**Fig. 2:**
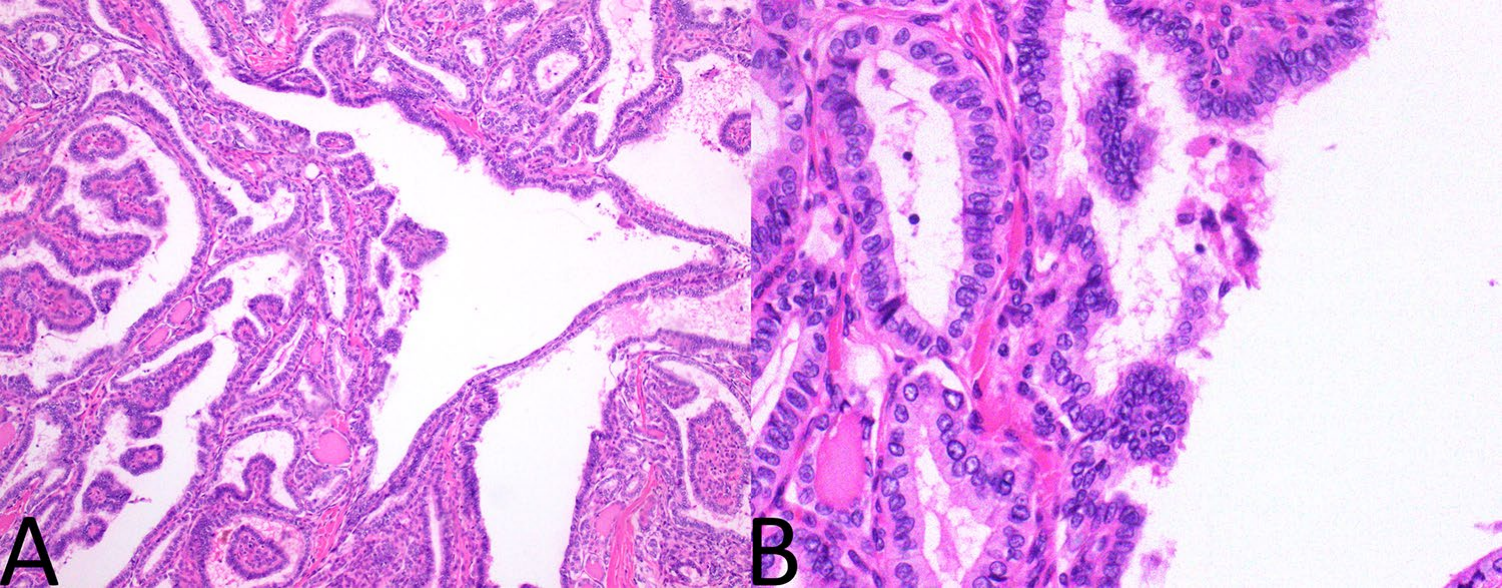
Papillary carcinoma with some tall cell features. **A** Papillary and follicular architecture (H&E, 100x). **B** Cells demonstrating nuclear features of papillary carcinoma with cells twice as high as they are wide (H&E, 400x)

**Fig. 3:**
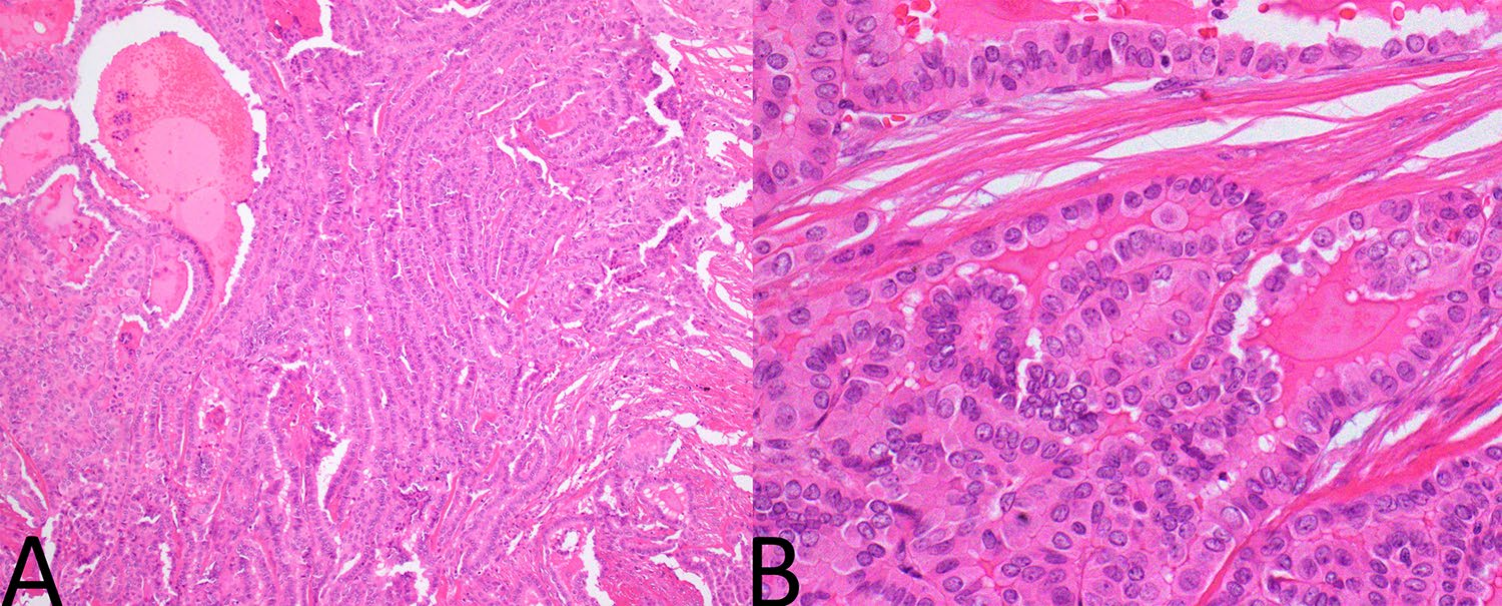
Papillary carcinoma with classical tall cell features. **A** Classic ‘tram tracking’ architecture with tubulopapillary structures arranged in back to back fashion (H&E, 100x). **B** Cells demonstrating papillary nuclear features with abundant cytoplasm at least 3 times as tall as wide (H&E, 400x)

A single representative haematoxylin and eosin slide (H&E) from each tumour was examined by an experienced endocrine pathologist (JT) who was blinded to all other clinical and pathological features at the time. The tumours were assessed for two factors—both the degree of tallness of the tall cells and the proportion of the tumour that demonstrated tall cells. That is, PTCs were divided into those with no significant tall cell component (defined as less than 30% tall cells) (Fig. 1); those with a tall cell component that was more than two times as tall as wide in at least 30% of cells (Fig. 2); and those with a tall cell component that was more than three times as tall as wide in at least 30% of cells (Fig. 3). The PTCs with a tall cell component were than divided into those comprised of 30 to 50% tall cells and those with > 50% tall cells.

A subset of the 20 most recent cases in the series was then retrieved, and the whole case was reviewed by the same pathologist, after a washout period of 12 months following the initial screening. The cases were selected because they were the most recent excisions in the series, and the slides were available for review. The pathologist selecting the series was not aware of the initial scores of these 20 cases due to the long-time delay (12 months), in a large series, between the initial screening and the whole case retrieval. At the time of re-screening the pathologist was blinded to the initial results and all clinical and outcome data. The tumours were re-evaluated for degree of tallness and proportion of the tumour with tall cells. The results were compared to the original evaluation, and a kappa score was calculated based on the presence or absence of any tall cell features.

According to the current ATA guidelines [10], criteria for low-risk papillary thyroid carcinoma include the absence of tall cell features, absent local or distant metastases, macroscopic complete excision, absent invasion of loco-regional structures, absent vascular invasion, and clinical N0 or ≤ five N1 nodal micrometastases. We therefore assessed the cohort as to whether they were ‘ATA guidelines low-risk-like’ (IE: fulfilled all criteria for low risk except absence of tall cell features) and ATA intermediate or high-risk-like (again defined using ATA criteria except tall cell definition).

Statistical analysis was performed using SPSS software (V25). Kaplan–Meier curve and log-rank test were used for survival analysis with disease free survival (DFS) being considered the main endpoint. A *P*-value of < 0.05 was considered significant. This study was approved by the Northern Sydney Local Health District Human Research Ethics Committee (ref: LNR 1312-417 M).

## Results

The results are summarized in Tables 1, 2, 3. The combined pathological and clinical database searches returned 561 PTCs. Of these, representative slides of tumour were sourced and reviewed in 544 cases but only 487 of those had sufficient follow up data. Of those, 38 PTCs (7.8%) were found to have TC-PTC as defined by the WHO 2022 system. That is, cells 3 times as high as wide in more than 30% of the tumour (Fig. 3). 144 (30%) PTCs had cells 2 times as high as wide in more than 30% of the tumour. 343 PTCs (70%) were considered to be negative for tall cell morphology by any criteria (that is less than 30% tall cells). 33 cases (7.0%) had tall cell morphology with cells 3 times high as wide in more than 50% of the tumour.

**Table 1:** Univariate analysis of prognostic factors for disease free survival

**Variable**	**Number (%) (*N* = 487)**	Median DFS (months)*	Mean DFS (months)*	**Univariate analysis**^**a**^		
				**HR**	**95% CI**	***p*-value**
**Age at diagnosis (years)**						
Median 47 (range 11–88)						
< 47	253 (52)	MNR	469	1		
> 47	234 (48)	226	255	1.649	0.854–3.184	0.136
**Gender**						
Female	365 (75)	384	305	1		
Male	122 (25)	MNR	334	2.045	1.047–3.995	**0.036**
**Lymph node metastasis**						
Absent	307 (63)	384	336	1		
Present	180 (37)	MNR	191	3.900	1.988–7.654	** < 0.0001**
**Proportion 2 × tall cells (%)**						
< 30	343 (70)	384	339	1		
> 30	144 (30)	MNR	208	2.548	1.323–4.907	**0.005**
**Proportion 2 × tall cells (%)**						
< 50	387 (79)	384	349	1		
> 50	100 (21)	193	176	2.440	1.218–4.885	**0.012**
**Proportion 3 × tall cells (%)**						
< 30	449 (92)	384	331	1		
> 30 (WHO TC-PTC)	38 (8)	MNR	178	2.114	0.819–5.456	0.122
**Proportion 3 × tall cells (%)**						
< 50	454 (93)	384	330	1		
> 50	33 (7)	MNR	184	1.877	0.660–5.336	0.237

Values in bold indicate statistical significance (P < 0.05)

*p*-values are obtained using chi^2^ test

*HR* hazard ratio, *CI* confidence interval, *hpf* high power field, *MNR* median not reached

^*^Disease-free survival calculated using Kaplan–Meier method

^a^Cox regression model

**Table 2:** Correlations between cell height and disease-free survival

	**All cases (*N* = 487)**						**Low-risk cases (*N* = 214)**						**Intermediate/high-risk cases (*N* = 273)**					
	**Number (%)**	**Median DFS (months)**	Mean DFS (months)*	**HR^**	**95% CI**	***p*-value**	**Number (%)**	**Median DFS (months)**	Mean DFS (months)*	**HR**^**a**^	**95% CI**	***p*-value**	**Number (%)**	**Median DFS (months)**	Mean DFS (months)*	**HR**^**a**^	**95% CI**	***p*-value**
**2 × cutoff (%)**																		
** < 30**	343 (70)	384	339	1			178 (83)	384	304	1			165 (60)	MNR	411	1		
** > 30**	144 (30)	MNR	208	2.548	1.323–4.907	**0.005**	36 (17)	193	210	5.617	1.241–25.425	**0.025**	108 (40)	MNR	182	1.529	0.735–3.183	0.256
**2 × cutoff (%)**																		
** < 50%**	387 (79)	384	349	1			189 (88)	384	316	1			198 (73)	MNR	412	1		
** > 50%**	100 (21)	193	176	2.440	1.218–4.885	**0.012**	25 (12)	193	174	10.750	1.793–64.445	**0.009**	75 (27)	MNR	176	1.512	0.688–3.325	0.303
**3 × cutoff (%)**																		
** < 30%**	449 (92)	384	331	1			207 (97)	384	299	1			242 (89)	MNR	405	1		
** > 30%**	38 (8)	MNR	178	2.114	0.819–5.456	0.122	7 (3)	34	71	59.301	8.265–425.503	** < 0.0001**	31 (11)	MNR	188	0.841	0.254–2.783	0.776
**3 × cutoff (%)**																		
** < 50**	454 (93)	384	330	1			208 (97)	384	299	1			246 (90)	MNR	403	1		
** > 50**	33 (7)	MNR	184	1.877	0.660–5.336	0.237	6 (3)	34	71	59.301	8.265–425.503	** < 0.0001**	27 (10)	MNR	199	0.647	0.154–2.727	0.553

Values in bold indicate statistical significance (P < 0.05)

*p*-values are obtained using chi^2^ test

*HR* hazard ratio, *CI* confidence interval, *MNR* median not reached

^*^Disease-free survival calculated using Kaplan–Meier method

^a^Cox regression model

**Table 3:** Multivariate analyses of various diagnostic criteria for TC-PTC and disease-free survival

	**All cases (*N* = 487)**			**Low-risk cases (*N* = 214)**			**Intermediate/high-risk cases (*N* = 273)**		
	**HR^**	**95% CI**	***p*-value**	**HR^**	**95% CI**	***p*-value**	**HR**^**a**^	**95% CI**	***p*-value**
**Proportion 2 × tall cells (%)**									
< 30	1			1			1		
> 30	1.770	0.829–3.776	0.140	9.106	0.886–93.568	0.063	1.371	0.622–3.021	0.434
**Proportion 2 × tall cells (%)**									
< 50	1			1			1		
> 50	1.778	0.814–3.887	0.149	13.106	1.016–169.101	**0.049**	1.539	0.674–3.512	0.306
**Proportion 3 × tall cells (%)**									
< 30	1			1			1		
> 30 (WHO TC–PTC)	0.652	0.178–2.391	0.519	98.868	4.253–2298.412	**0.004**	0.520	0.119–2.268	0.384
**Proportion 3 × tall cells (%)**									
< 50	1			1			1		
> 50	0.719	0.197–2.619	0.617	98.868	4.253–2298.412	**0.004**	0.583	0.134–2.525	0.470

Values in bold indicate statistical significance (P < 0.05)

*p*-values are obtained using chi^2^ test

*HR* hazard ratio, *CI* confidence interval

^a^Cox regression model controlling for lymph node metastasis, vascular invasion, and tumour size

In the 20 cases where the full case was reviewed, there was good concordance between the original score on a single slide, and the score given 12 months later after reviewing the whole case. 5 cases of 3 × cell height were included, 5 cases of 2 × cell height were included, and the remaining 10 had no tall cell features. 9 cases of more than 50% proportion were included, 1 case of 30–50% was included and 10 cases of no tall cell features were included. 16 of the 20 cases were completely concordant for degree of tallness, and 17 of 20 were completely concordant for proportion of tall cells. 18 of 20 cases were concordant for any tall cell features. A kappa score of 0.79 was reached.

On univariate analysis, there was a statistically significant difference in disease-free survival (DFS) in PTCs with 30% or more neoplastic cells 2 times as tall as wide (*p* = 0.005). However, when TC-PTC was defined as cells 3 times as tall as wide in 30% of more the tumour (the WHO 2022 criteria), TC-PTC failed to reach statistical significance (*p* = 0.122).

When tall cells were defined as either two times or three times as high as wide, the proportion of tall cells (30–50% vs. > 50%) showed a statistically significant difference in DFS in 2 × height (*p* = 0.005 and *p* = 0.012) but not in 3 × height (*p* = 0.122 and *p* = 0.237). That is, in univariate analysis, the greater the proportion of the tumour that was tall cell, the poorer the outcome when the 2 × height cut off was used but not when the 3 × height cut off was used.

In Table 2, the tumours are subclassified into ‘ATA low-risk-like’ and ‘ATA intermediate to high-risk-like’. In tumours classified as otherwise low risk according to ATA guidelines, a diagnosis of TC-PTC (as per WHO criteria) was associated with a significantly shorter DFS (median 34 vs. 384 months, mean 71 vs. 299 months, *p* < 0.0001). Similarly, in tumours classified as low risk, cell height 2 × width using 30% was also associated with significantly shorter DFS (median 193 months and mean 210 months, *p* = 0.025), but DFS was much better compared to TC-PTC as per WHO criteria. In contrast, a diagnosis of TC-PTC in intermediate/high-risk tumours by WHO criteria had no significant impact on DFS (medians not reached, mean 188 vs. 405 months, *p* = 0.776). Similarly, there was no association between DFS and TC-PTC in intermediate/high-risk tumours using any of the other potential criteria.

**Fig. 4:**
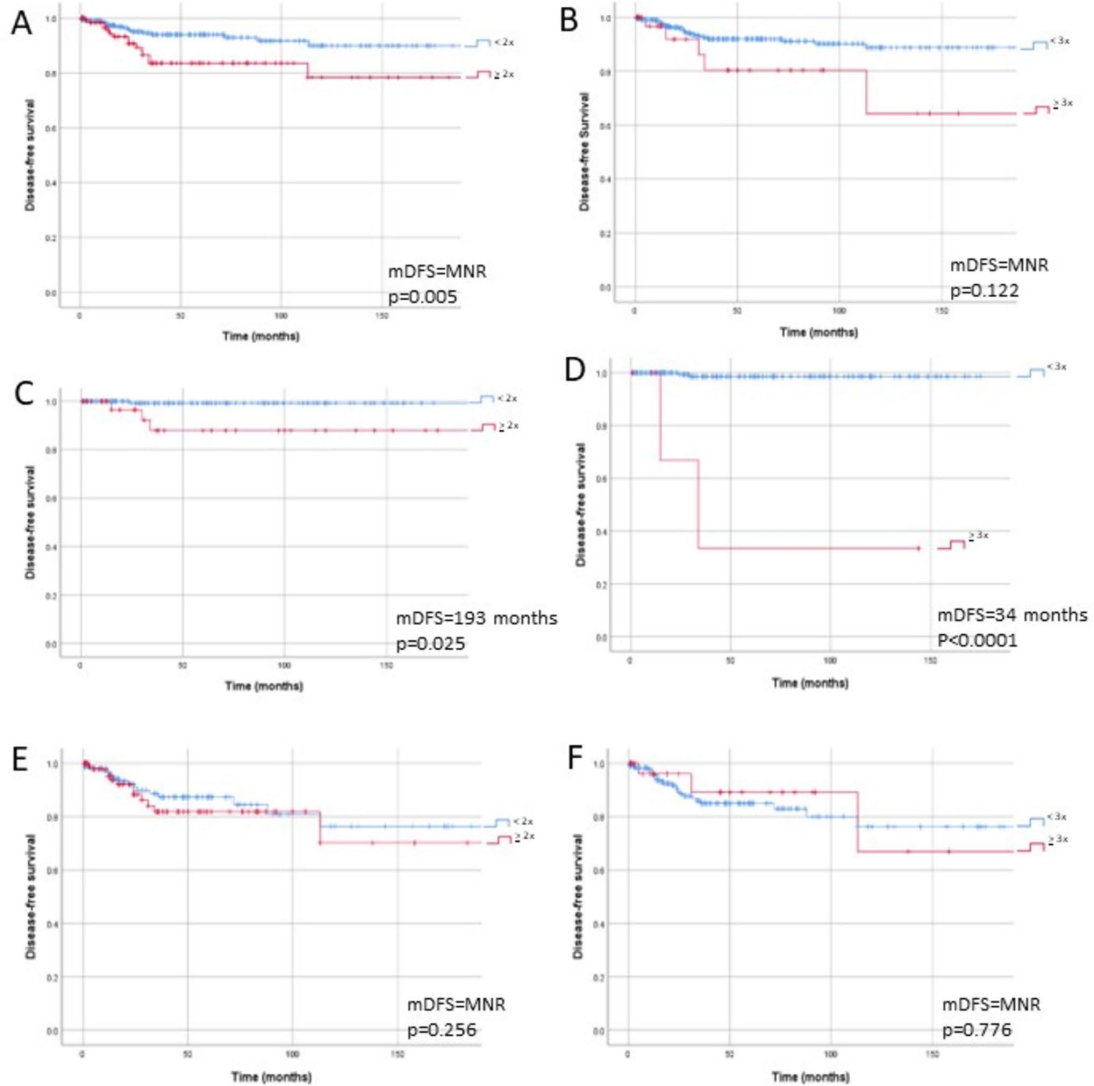
Kaplan Meier curves for cell height and median disease-free survival (mDFS): **A** all cases 2 × cell height; **B** all cases 3 × cell height; **C** low-risk cases 2 × cell height; **D** low-risk cases 3 × cell height; **E** high-risk cases 2 × cell height; **F** high-risk cases 3 × cell height. MNR = median not reached

In Table 3, a multivariate analysis was applied controlling for lymph node metastasis, vascular space invasion, and tumour size. When applied across the entire cohort, no criteria for tall cell subtype had statistical significance. When separated into low risk and high-risk-like stratification, the high-risk-like subgroup maintained this lack of statistical significance. However, the low-risk-like subgroup demonstrated statistical significance for reduced disease free survival when the proportion of tall cells was greater than 50% of the tumour and either 2 or 3 times cell height (*p* = 0.049 and *p* = 0.004). Statistical significance was also maintained with the WHO criteria of 3 times height in > 30% of cells (*p* = 0.004) (Fig. 4). That is, in multivariate analysis TC-PTC was only statistically significantly associated with adverse outcomes in tumours which were otherwise low risk.

## Discussion

There is no doubt that the presence of tall cell components is associated with adverse outcomes in PTC. However, we emphasize that tallness is a spectrum with different significances in different settings. For example, in simple univariate analysis, the greater the proportion of tall cells portended a worse when the 2 times cut off was used but not with a 3 times cut off. We note that our study includes cases that are both *RAS* driven and *BRAFV600E* driven, as this was an unselected series. As such it is likely that the PTCs which do not have tall cell features are more likely to be *RAS* driven tumours, and those that show any tallness are more likely to be *BRAFV600E* driven; and this difference in the underlying molecular events may account for some of the clinical significance of 2 times height cut off [36–38].

Secondly, and most importantly, we emphasize that despite these strong associations in univariate analysis, the actual clinical significance of TC-PTC and whether major management decisions should be made on the basis of tallness alone is much more difficult to define. This is because of the clear relationship between the presence of tallness and other adverse histological features which are already considered in clinical decision-making. Indeed, in this study in a multivariate model that included age, size, vascular invasion, and lymph node metastasis, TC-PTC by WHO criteria lost statistical significance for DFS (*p* = 0.519) when the cohort was considered as a whole. However, on subgroup analysis when tumours were separated into otherwise low-risk or high-risk PTCs, the presence of TC-PTC by three criteria (2 × and 50%, 3 × and 30%, and 3 × and 50%) was statistically significant in low-risk patients (*p* = 0.049, *p* = 0.004, and *p* = 0.004, respectively) and the criteria of 2 × and 30% just failed to reach statistical significance (*p* = 0.063). In fact, none of the different criteria for TC-PTC maintained clinical significance for DFS in the intermediate/high-risk patients.

Therefore, we conclude that, given the strong associations between TC-PTC and other adverse prognostic features, the diagnosis of TC-PTC only seems to have clinical significance in tumours which would otherwise be classified as ATA low risk.

This has significant clinical implications. According to the current ATA guidelines [10] in addition to the absence of tall cell features, criteria for low-risk papillary thyroid carcinoma include the absence of local or distant metastases, macroscopic complete excision, absent invasion of loco-regional structures, absent vascular invasion, and clinical N0 or ≤ five N1 nodal micrometastases. Our data clearly supports the ATA approach that the presence of tallness should be an exclusion criteria for low-risk disease. However, it also suggests that the presence of tallness lacks clinical significance in intermediate or high-risk disease. Therefore, we would caution against offering more aggressive treatment to a patient with TC morphology compared to classical or other subtypes of PTC, if the tumour would already be considered intermediate or high risk by ATA guidelines.

Noting that the WHO 2022 criteria (30% of cells three times as tall as wide) does not have clinical significance over all cases in our cohort, we question the clinical value of mentioning tallness (or at least changing clinical treatment on the basis of tallness) in tumours which have other adverse features. Given that the diagnosis of TC-PTC has the most clinical significance in otherwise low-risk patients, the next question is what cut-offs should be applied. In our data (specifically Table 3), a diagnosis of tall cell subtype should only be applied to an otherwise low-risk tumour, if either it fulfils the current WHO criteria of 3 times the cell width in more than 30% of cells or if the cells are twice the height in more than 50% or cells. A diagnosis of TC-PTC should not be rendered if the cells are two times as tall as wide in only 30% of the cells.

Whilst our study is robust and is strengthened by the fact that most of the TC-PTCs we describe were not prospectively identified as such and therefore different treatments would not have affected outcome, it is not without limitations. Firstly, we only reviewed one slide from each tumour for most of the patients. It is well established that the proportion of tall cell features can be focal and highly variable in a single tumour and it could be that some tumours had a greater or less component of tall cells which could have changed our final diagnosis. However, we note that we also demonstrated that on review of the whole tumour in a subset of 20 cases there was excellent concordance between 1 representative slide and the whole tumour (kappa score = 0.79). Therefore, although we accept that screening only 1 representative slide for most of the cohort remains a weakness of the study, there is data to suggest that our findings are generalizable.

Secondly, although all cases were scored by an experienced endocrine pathologist who demonstrated good concordance with the original classification after a 12-month washout period, we did not assess the interobserver concordance for the diagnosis of TC-PTC. We stress that TC-PTC is a very subjective diagnosis which is highly susceptible to interobserver variability which should also be considered in the context of our findings. As such, we caution the use of the tall cell subtype in general unless the diagnosis is made by an experienced endocrine pathologist, or concordance is achieved by multiple pathologists prior to diagnosis.

With the advent of the differentiated high-grade thyroid carcinoma (DHGTC) classification in the new WHO classification, another limitation remains in that some of the tumours included in this series may in fact be DHGTC and therefore should not be included [1]. This is especially true as the criteria for this diagnosis includes a mitotic count greater than or equal to 5 or tumour necrosis which may not be seen on 1 representative slide [1]. However, in our experience, tumour necrosis and high mitotic counts are readily identifiable across any part of an aggressive thyroid neoplasm and are usually not confined to 1 area. This means that our cohort is unlikely to have these cases included, although we fully accept we cannot be definitive about this.

This study is highly relevant in the context of digital microscopy. Although we were limited by the use of light microscopy, as this is still in widespread use in Australia, digital microscopy is rapidly becoming mainstream in many parts of North America and Europe. This technology would enable pathologists to accurately estimate the percentage of tall cells within a tumour and indeed artificial intelligence may also have a role in estimating the tall cell components of a tumour. This may be particularly important in tumours with only very minor tall cell components (< 10%) which were not considered in this study [39].

Other valuable data not able to be included in this study includes the molecular profile of the tumours. It is well known that TC-PTC is associated with BRAFV600E mutation [29]. This is relevant in the context of BRAFV600E mutant disease being more likely to be radioactive iodine refractory and therefore complicating the treatment options of this group. Whilst the molecular status of these tumours would be of relevance to the results, the testing of this cohort is beyond the limits of this study due to the age of the included cases and costs. However, it would be data worth extracting in future endeavours, especially given the relative ease of extracting molecular data in many modern laboratories.

We conclude tallness is a spectrum where both the percentage of cells that are tall and their height have different clinical significances in different settings. TC-PTC is strongly associated with other easily identified adverse prognostic factors such as size and lymphovascular space invasion and therefore the diagnosis of TC-PTC in tumours that are otherwise intermediate or high risk has little clinical significance. Our data indicate that the primary clinical significance of TC-PTC is for tumours which are otherwise low risk, and therefore, our data supports the ATA criteria of considering TC-PTC an exclusion criterion for low-risk disease. For this reason, if the diagnosis of TC-PTC is to be proffered, we recommend restricting it to criteria which have proven clinical significance in the otherwise low-risk cohort. Whilst an argument could be made to include tumours where more than 50% of the tumour is composed of cells which are twice times as tall as they are wide, this approach is not in widespread use. Therefore, we endorse the current WHO criteria of restricting the diagnosis of TC-PTC to PTCs in which more than 30% of the tumour is composed of cells which are 3 times tall as wide.

In summary, our data supports the current WHO definition of TC-PTC but makes the important point that the diagnosis only seems to have clinical significance in PTCs that would otherwise be considered low risk.

## Data Availability

Datasets are stored in a spreadsheet (excel) and in an SPSS file both of which are securely stored on a central password protected computer in a locked office. The slides are stored in a locked storage cupboard in a locked office.
